# Atrial epicardial adipose tissue abundantly secretes myeloperoxidase and activates atrial fibroblasts in patients with atrial fibrillation

**DOI:** 10.1186/s12967-023-04231-2

**Published:** 2023-06-06

**Authors:** Eva R. Meulendijks, Rushd F. M. Al-Shama, Makiri Kawasaki, Benedetta Fabrizi, Jolien Neefs, Robin Wesselink, Auriane C. Ernault, Sander Piersma, Thang V. Pham, Connie R. Jimenez, Jaco C. Knol, Wim J. P. van Boven, Antoine H. G. Driessen, Tim A. C. de Vries, Britt van der Leeden, Hans W. M. Niessen, Onno J. de Boer, Sébastien P. J. Krul, Joris R. de Groot

**Affiliations:** 1 Amsterdam UMC, Heart Center, Department of Clinical and Experimental Cardiology and Cardiothoracic Surgery, University of Amsterdam, Amsterdam, The Netherlands; 2 Amsterdam Cardiovascular Sciences, Heart Failure and Arrhythmias, Amsterdam, The Netherlands; 3 Amsterdam UMC, VU Medical Center, Department of Medical Oncology, VU university, Amsterdam, The Netherlands; 4 Amsterdam UMC, Department of Pathology, University of Amsterdam, Amsterdam, The Netherlands; 5 Department of Cardiology, Rijnstate Hospital, Arnhem, The Netherlands; 6 Amsterdam UMC, Infection & Immunity, University of Amsterdam, Amsterdam, The Netherlands; 7 Department of Cardiology, Isala Heart Centre, Zwolle, The Netherlands

## Abstract

**Background:**

Epicardial adipose tissue (EAT) secretome induces fibrosis. Fibrosis, primarily extracellular matrix (ECM) produced by fibroblasts, creates a substrate for atrial fibrillation (AF). Whether the EAT secretome from patients with AF activates human atrial fibroblasts and through which components, remains unexplored.

**Research aims:**

(a) To investigate if the EAT secretome from patients with versus without AF increases ECM production in atrial fibroblasts. (b) To identify profibrotic proteins and processes in the EAT secretome and EAT from patients with, who will develop (future onset), and without AF.

**Methods:**

Atrial EAT was obtainded during thoracoscopic ablation (AF, n = 20), or open-heart surgery (future onset and non-AF, n = 35). ECM gene expression of human atrial fibroblasts exposed to the EAT secretome and the proteomes of EAT secretome and EAT were assessed in patients with and without AF. Myeloperoxidase and neutrophil extracellular traps (NETs) were assessed immunohistochemically in patients with paroxysmal, persistent, future onset, and those who remain free of AF (non-AF).

**Results:**

The expression of COL1A1 and FN1 in fibroblasts exposed to secretome from patients with AF was 3.7 and 4.7 times higher than in patients without AF (p < 0.05). Myeloperoxidase was the most increased protein in the EAT secretome and EAT from patients with versus without AF (FC 18.07 and 21.57, p < 0.005), as was the gene-set neutrophil degranulation. Immunohistochemically, myeloperoxidase was highest in persistent (FC 13.3, p < 0.0001) and increased in future onset AF (FC 2.4, p = 0.02) versus non-AF. Myeloperoxidase aggregated subepicardially and around fibrofatty infiltrates. NETs were increased in patients with persistent versus non-AF (p = 0.03).

**Conclusion:**

In AF, the EAT secretome induces ECM gene expression in atrial fibroblasts and contains abundant myeloperoxidase. EAT myeloperoxidase was increased prior to AF onset, and both myeloperoxidase and NETs were highest in persistent AF, highlighting the role of EAT neutrophils in the pathophysiology of AF.

**Supplementary Information:**

The online version contains supplementary material available at 10.1186/s12967-023-04231-2.

## Introduction

Atrial fibrillation (AF) is the most common sustained cardiac arrhythmia worldwide. AF is associated with diminished quality of life, heightened stroke risk, and increased mortality rates [11, 19, 23]. Consequently, AF imposes a high and escalating burden on our healthcare system [4]. AF is often accompanied by atrial structural remodeling, primarily fibrosis, which results from excessive extracellular matrix (ECM) production by fibroblasts. However, specific therapeutic targets to halt or prevent structural remodeling are lacking, as the specific drivers of atrial fibrosis in AF are largely unidentified. The metabolically active epicardial adipose tissue (EAT) may contribute to atrial remodeling [14, 35]. The volume of EAT has been identified as an independent risk factor for the onset, severity, and recurrence of AF [7, 14, 27]. EAT from AF patients secretes more extracellular vesicles that hold increased levels of pro-inflammatory and fibrotic proteins,compared to patients without AF, leading to increasead ventricular fibrosis and reduced action potential duration in rats This suggests a causal effect of the EAT vesicles on arrhythmogenicity [36]. It is largely unexplored whether exposure to the EAT secretome from relatively healthy patients with AF induces more fibrosis compared to those without AF, and through which components. To this end, we examined the differential expression of ECM genes in human atrial fibroblasts exposed to the EAT secretome of patients with and without AF. To provide insight into proteome differences between patients with and without AF we performed untargeted explorative proteomic analyses of both the EAT secretome and EAT. We previously employed a similar methodology to explore processes involved in atrial substrate formation in atrial myocardium of these same patients [20]. Here, we histologically validated and localized the identified proteins and assessed their presence prior to the onset of AF.

## Materials and methods

### Patient recruitment and exclusion criteria

For this study, we included two groups of patients: those with AF undergoing stand-alone thoracoscopic ablation (n = 20, from the MARK AF registry NL5006901819), and those without a history of AF undergoing coronary bypass graft/valve surgery (n = 35, from the PREDICT-AF study, NCT03130985) [42]. As part of both studies, the left atrial appendage (LAA) was excised and used for molecular and histopathological analyses. The 150 included patients in the PREDICT-AF study were free from a history of AF at baseline, confirmed by thorough preoperative rhythm monitoring, and underwent either coronary bypass or valve surgery between 2015and2018. They were followed for separate assessment of the development of postoperative AF (≤ 30 days) and for future onset AF > 30 days. rRelevant exclusion criteria for both studies were systemic inflammation, endocarditis or pericarditis, and left ventricular ejection fraction of < 35%. AF classification was based on the ESC guidelines [18]. All patients received a prespecified follow-up with a 24-h Holter and ECG at 1, 6, 12 months to assess the development of AF [42].

We matched patients per experiment. For the fibroblast and proteomics studies, we first randomly selected EAT secretome and EAT samples from patients without AF, and matched them to patients with AF by sex and BMI. For the histopathological studies, all patients who developed AF after 30 days up to 1 year follow-up (referred to as future onset AF, n = 15) were included and matched for age, BMI, sex, diabetes, and CHADSVASc Score to patients who remained free from postoperative and future onset AF. Patients who developed cancer or died during the first year were not included for analysis.

It is worth noting that the transcriptome of patients with new onset AF from the published PREDICT-AF study was significantly altered compared with to those who remained free from AF, while clinical characteristics were not associated with AF onset. In a seperate study, we showed that changes in atrial gene expression of ECM genes in particular differed in an ordinal manner between patients without (PREDICT-AF) and those with paroxysmal and persistent AF (MARK-AF), independently of clinical characteristics [41]. We therefore assume that differences in clinical characteristics that were present between patients without AF (undergoing cardiothoracic surgery) and patients with paroxysmal or persistent AF (undergoing stand-alone thoracoscopic ablation) did not confound the findings Both the MARK AF registry and the PREDICT AF study were approved by the Amsterdam University Medical Center medical ethical committee. All participants provided written informed consent.

### Patient material

Upon excision, theEAT was dissected and separated directly from the myocardium of the LAA. Pieces of the EAT were either immediately snap-frozen at − 80 °C for EAT proteomics and ribonucleic acid (RNA) isolation, stored in formaldehyde for histopathological analysis, or directly placed in phosphate-buffered saline (PBS) for secretome collection. For the latter, the EAT was cut into cubes of roughly 1 mm^3^ (± 20 per sample). These cubes were washed thrice for 5 min (15 min total) to remove blood and other contaminants. Individual cubes were incubated in 100 µl PBS on a thermo-shaker at 250 rotations per minute at 37 °C for 1 h and subsequently centrifuged to remove cell and tissue debris. The secretome was harvested and snap-frozen in liquid nitrogen and stored at − 80 °C.

### ECM gene expression of human atrial fibroblasts cultured with the EAT secretome

Normal heart atrial cardiac fibroblasts from LONZA (Cat#. CC-2903) were cultured in the growth medium FBM™ Basal Medium (CC-3131) with FGM™-3 SingleQuot Supplements (CC-4525) containing FBS, Human Fibroblastic Growth Factor-B, Gentamicin sulfate-Amphotericin, and insulin. The secretome of 14 patients with and 4 without AF was added to the fibroblasts, which were first cultured for 6 h in 0.5%FBS culture medium. The volume of the EAT secretome was corrected for the weight of the EAT sample after secretome harvesting. After 48 h of incubation, the gene expressions of COL1A1 and FN1 were measured. The potential between-session multiplicative variation was corrected using factor correction, i.e., division of the data in each session with a session-specific correction factor [34]. Fibroblasts of passages three to five were used for our experiments. The fibroblast traits were assured in these passages according to the manufacturer’s protocol. For RNA isolation, samples were cut into 50–150 mg pieces, and RNA was isolated using Trizol (Invitrogen™, Cat# 15596018) according to the manufacturer’s protocol and assessed by the nanodrop (Thermo Scientific One Spectrophometer). cDNA was synthesized using the SuperScript™ II reverse transcriptase protocol (Invitrogen™, Cat# 18064022), after which qPCR was performed using the SYBR Green PCR Kit (Roche, Cat# 04707516001) on the LightCycler 480 (Roche). Genes were quantified by performing linear regression analysis using LigRegPCR [30] software and then normalized to human hypoxanthine phosphoribosyltransferase (*hHRPT*). The gene primer sets used for amplification are displayed in Additional file 1: Table S1.

### Proteomics—untargeted mass spectrometry (LC–MS/MS) on EAT and EAT secretome

Proteins and their peptides were extracted from both the EAT and EAT secretome of 3 patients with and 3 without AF by liquid chromatography-tandem mass spectrometry using an Ultimate 3000 Nano LC–MS/MS system (Dionex LC-Packings, Amsterdam, The Netherlands) and identified as described previously [20, 29]. Specific to this study, secretome extracted from ± 20 1 mm EAT cubes per sample were concentrated approximately 40 times using ultrafiltration devices (Amicon Ultra-4 Centrifugal Filter Unit, millipore, UFC801024), and EAT samples were cryo-milled and solubilized before protein denaturation and fixation (gels: Additional file 1: Fig. S1). For protein identification, MS/MS spectra were searched against the Swiss-Prot human FASTA file (canonical and isoforms, downloaded March 2017, 42161 entries) using MaxQuant version 1.5.4.1.[9]. Proteomes from EAT and EAT secretome were uploaded to the ProteomeXchange Consortium via the proteomics identification database PRIDE with accession number PXD013230.

### Proteomics—data management and data mining

The 50 most increased and decreased proteins extracted from the EAT secretome and EAT were listed based on significance (b-binomial p-value) and relative expression (fold-change). Data were displayed in volcano plots for a parallel assessment of significance and relative expression. Gene-set enrichment analysis (GSEA) for biological processes (Baderlab.org, gene set February 2022) was conducted on the protein list pre-ranked by both protein significance and relative expression, with resultsvisualized in Cytoscape version 3.9 [32, 37, 45]. For in-depth data analysis and reduction, the MCL clustering and EnrichmentMap applications were used. In addition, heatmaps and Venn diagrams were created by online applications from Heatmapper and bioinformatics [3].

### Histopathological quantifications in AF, future onset AF and non-AF patients

For validation and localization of identified proteins, paraffinized LAAs of 10 persistent, 6 paroxysmal, 15 future onset AF, and 14 non-AF patients were sectioned into 5 μm slices and histopathologically assessed. All quantifications were performed on each sections total tissue area in Qupath 3.0 [5]. Sections were stained immunohistochemically with an antibody against MPO (DAKO A0398, dilution 1:4000), visualized using DAB (Immunologic BrightDAB BS04), and digitizedat a 40 × magnification, 0.25 μm/pixel (Philips IntelliSite Ultra Fast Scanner). MPO was quantified using a neural network-based MPO pixel classifier (Additional file 1: Fig. S2). Separate annotations were made for the total EAT, defined as the region from the outer epicardial border to the myocardium, and for the myocardium region, which included the endocardium and marginally present adipose tissue. The subepicardial region was defined as a 50 μm distance from the outside epicardial border. Neutrophil extracellular traps (NETs) were visualized by immunofluorescence and were defined as single MPO positive cells without macrophage (CD14) positivity, localized within web-like structures of histone H3. Only extravascular (identifiable by CD31) NETs were quantified. Antibodies against MPO (DAKO A0398, dilution 1/400), CD14 (Abcam, ab181470, 1/100), histone H3 (citrulline R2 + R8 + R17, Abcam, ab5103, 1/100), CD31 (DAKO, M0823 clone JC70A, 1/200), and Hoechst (Thermofisher Scientific, 33342 solution, 1/1000) were used. Sections were digitized at a 20 × magnification, 0.5 μm/pixel (Vectra Polaris Automated Quantitative Pathology Imaging System). MPO and NET quantifications were repeated on 10 random samples by second assessors (R.A.S, and B.L) and third assessors (H.W.M.N) in the case of discrepancies.

### Statistical analysis

We used the beta-binomial test to evaluate differences in normalized spectral counts between AF and non-AF. Proteins were significantly differentially expressed when *p*-values were < 0.01, and were additionally subjected to significance testing after Benjamini-Hochberg (BH) correction, (which restricted the false discovery rate (FDR) to 0.25).We included only gene-sets with FDRq < 0.1 after performing weighted GSEA analysis. We assessed statistical significance between continuous normally distributed data using the unpaired samples t-test, non-normally distributed continuous and ordinal data using the Mann–Whitney u-test, and nominal variables using Fisher's exact test. For three-group comparisons, we used one-way ANOVA for continuous data and the chi-square test for categorical and nominal data. We tested inter-observer reliability using Pearson’s correlation. We expressed the data as mean ± SD, median ± [IQR], n(%), or other as specified. Statistical analyses were performed using SPSS (version 28).

## Results

### Patient characteristics

Table 1 presents the characteristics of patients with AF, future onset AF, and non-AF for all experiments combined. AF patients had a higher indexed left atrial volume (LAVI), had less diabetes, were younger, had a lower CHA_2_DS_2_-VASc score and white cell count, and had less vascular disease than patients without AF. Consequently, AF patients were less likely to receive statins and antiplatelet therapy than those without AF. Among patients without AF, those who developed future onset AF had similar characteristics compared to those who remained free from AF. For each experiment, most cardiovascular risk factors were equally distributed between patients with and without AF at baseline. For instance, in the proteomics studies specifically, 2 AF and 2 non-AF patients had diabetes, and 2 AF and 3 non-AF patients had vascular disease [20].

**Table 1:** Patient characteristics

	patient with AF		patients without AF					
variable	persistent AF n = 14, paroxysmal AF n = 6		future onset AF (1y after surgery) n = 15		non-AF, 1 year after surgery n = 20		p value: AF vs without AF	p value: future onset vs non-AF
Surgery type n (%)	Thoracos-copic AF ablation	20 (100)	CABG/AVR	14 (93), 1 (7)	CABG/AVR	18 (90), 2 (10)	N/A	
Male sex n (%)	19	(95.0)	12	(80)	17	(85)	0.30	0.6
Age [IQR]	59.5	[10.4]	73.6	[16.3]	69.1	[8.2]	< 0.01	0.4
BMI (± SD)	29.0	(3.4)	27.5	(3.6)	27.3	(3.0)	0.09	0.8
LAVI % [IQR]	41.9	[14.7]	30.3	[15]	25.8	[11]	< 0.01	0.9
CHA_2_DS_2_-VASc [IQR]	1	[2]	3	[2]	3	[1]	< 0.01	0.5
Hypertension n (%)	9	(45)	9	(60)	11	(55)	0.42	1.0
Diabetes n (%)	3	(15)	5	(30)	10	(50)	0.04	0.3
Vascular disease n (%)	3	(15)	13	(87)	19	(95)	< 0.01	0.6
Stroke n (%)	2	(10)	1	(7)	3	(15)	1	1.0
Congestive heartfailure n (%)	1	(5)	0	0	0	0	0.30	1.0
CRP [IQR]	1.4	[2.4]	1.8	[2.9]	1.4	[5]	0.45	0.6
Leukocytes, 10^–9^/L (± SD)	6.5	(1.4)	7.6	(1.5)	7.6	(2.1)	0.02	1.0
Thrombocytes, 10^–9^/L (± SD)	252.4	(39.5)	222	(53.1)	242	(41.5)	0.14	0.5
OAC n (%)	20	(100)	0	(0)	0	(0)	< 0.01	1.0
Antiplatelets n (%)	0	(0)	14	(93)	19	(95)	< 0.01	1.0
Statins n (%)	2	(10)	10	(66)	19	(95)	< 0.01	0.1

### EAT secretome from patients with AF induced ECM gene expression in human atrial fibroblasts

**Fig. 1:**
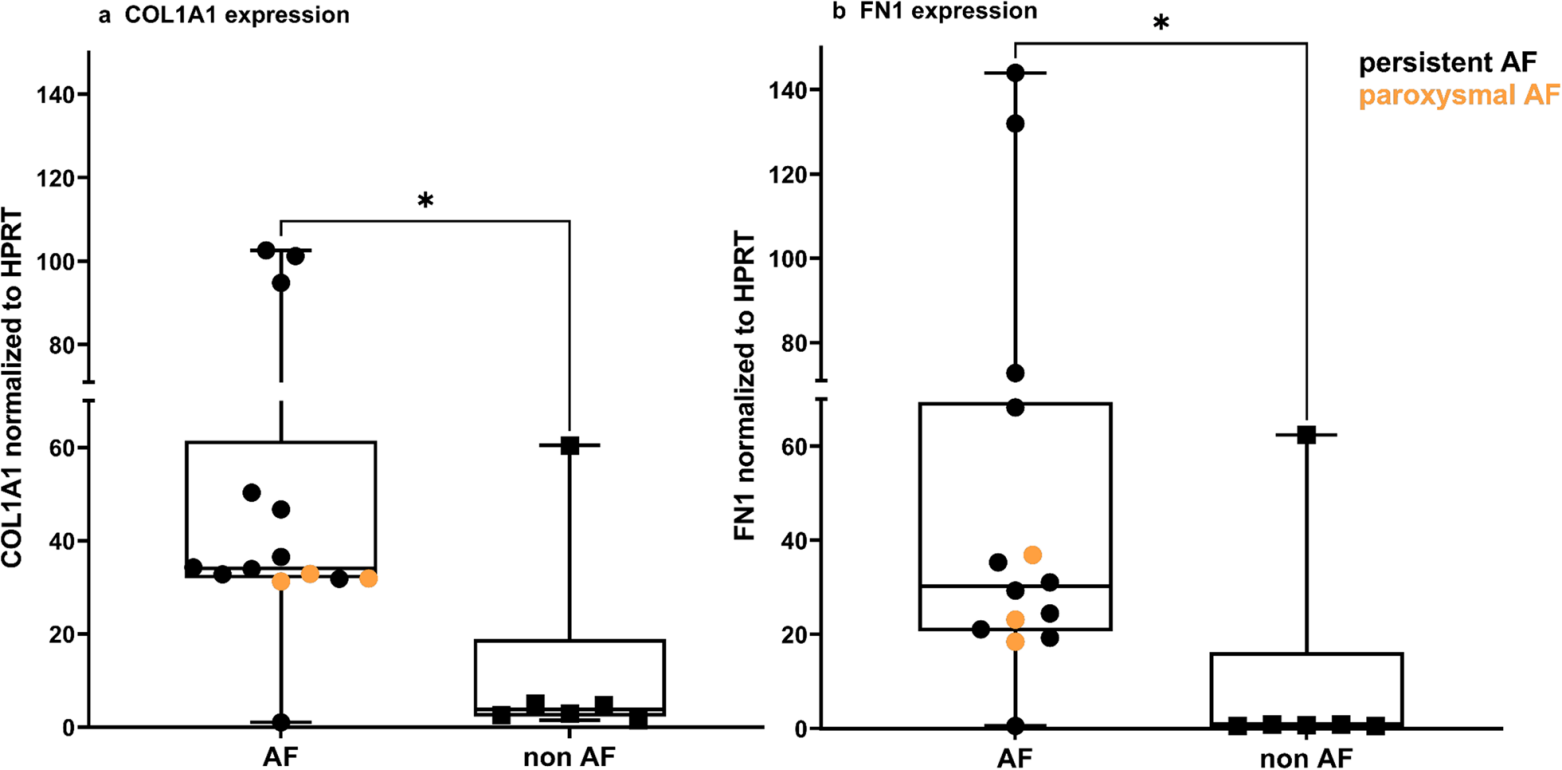
**a**, **b**. Atrial fibroblast ECM gene expression after exposure of EAT secretome from 11 persistent, 3 paroxysmal, and 6 patients without AF. a COL1A1, collagen type 1, alpha 1. b FN1, fibronectin. Paroxysmal AF patients are coloured in yellow. Bars represent median [IQR]. Results are corrected for batch affect. *p-value: <0.05

After exposure to EAT secretome, *COL1A1* and *FN1* expression in human atrial fibroblasts were 3.7 and 4.7 times higher, respectively, in patients with versus without AF (p = 0.03, p = 0.02), (Fig. 1a, b, Additional file 1: Table S2).

### Differentially expressed proteins in the EAT secretome and EAT

**Fig. 2:**
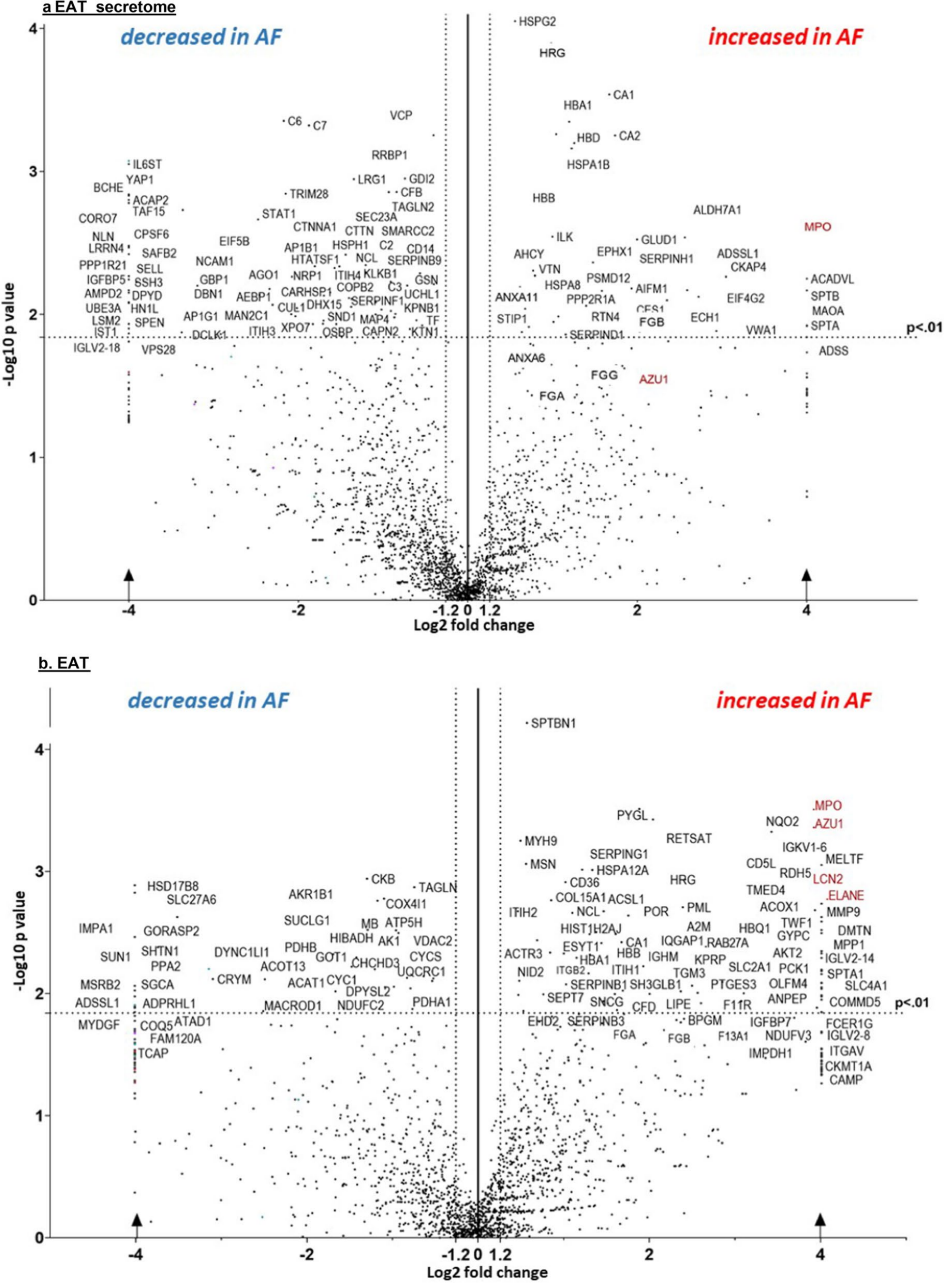
**a**, **b**. Identified proteins by LC/MS-MS in a. EAT secretome (2460), and b. EAT (2233), from patients with versus without AF. The log-transformed x-axis represent fold-change (FC), y-ax the significance. Dotted vertical lines indicate a 1.2 (FC), and the horizontal lines a p-value of 0.01. Proteins only expressed in patients with or without AF, or those with a FC of 4> are plotted on the cut-off Log2 fold-change of 4/-4 (arrows). Neutrophil proteins are colored red

In the proteome of EAT secretome and EAT, 2460 and 2233 unique proteins were identified, respectively (Fig. 2a, b). Unsupervised clustering revealed that the protein profiles of patients with and without AF could be distinguished in both the EAT secretome and the EAT (Additional file 1: Fig. S3a, b). The EAT secretome showed 83 differentially expressed proteins between patients with and without AF (p < 0.01, FC > 1.3 or < − 1.3) (Fig. 2a). Of these, proteins were significantly increased and 57 decreased in AF. Thirty-one proteins remained significantly differentially expressed after BH correction. In the EAT proteome, 98 proteins were differentially expressed between patients with and without AF (p < 0.01, or FC > 1.3 or < − 1.3) (Fig. 2b). Of these, 57 were increased and 41 were decreased in AF. After BH correction, 119 proteins remained significantly differentially expressed. The top 50 most differentially expressed proteins in the EAT secretome and EAT are listed in Additional file 1: Tables S3 and S4, respectively. Among the most increased proteins in both the proteomes of the EAT secretome and EAT were neutrophil proteins (Fig. 2a, b). MPO was the only significantly increased protein in the EAT secretome (FC 18.08, p = 0.003), EAT (FC 21.37, p = 0.005), and myocardium (including marginally EAT) (FC 8.51, p < 0.001) of patients with versus without AF (Additional file 1: Fig. S4). Hemostasis proteins such as histidine-rich glycoprotein (HRG) and to a lesser extent fibrinogen subunits, were increased in EAT secretome and EAT (HRG: FC 1.98, p < 0.001, FC 5.35, p = 0.001, FGA: FC 1,92, p = 0.04, FC2.53, p = 0.02, FGB: FC 4.01, p0.01, FC 2.48, p = 0.05, and FGG: FC 3.03, p = 0.04, FC 3.08, p = 0.01). ECM proteins such as collagen alpha-1 (XV) were also increased in EAT secretome and EAT (FC 2.40, p = 0.016, FC N/A, p = 0.004). Adiponectin expression was similar in AF versus non-AF patients in both EAT secretome and EAT (FC 1.10, p = 0.80, FC -1.97, p = 0.22), as were omentin (FC -1.14, p = 0.50, FC1.57, p = 0.34) and chemerin (only in EAT: FC 3.38, p = 0.49).

### Differentially expressed processes in the EAT Proteome

**Fig. 3:**
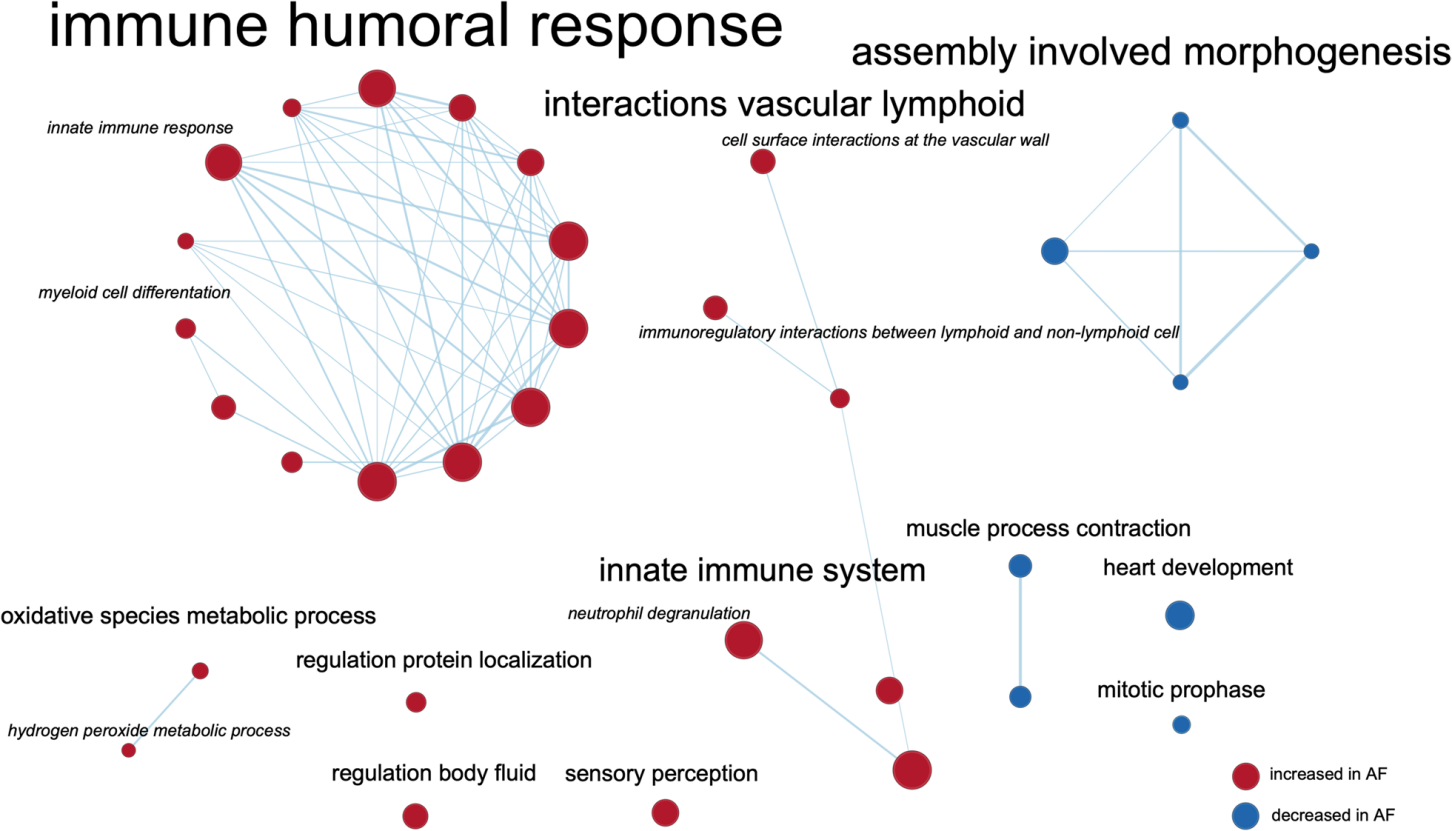
Gene set enrichment analysis of EAT. Nodes represent biological processes clustered based on protein similarity. Node-size indicates number of proteins annotated to that process. Only nodes with FDRq < 0.1 are presented. Connecting lines indicate protein similarity between two processes of at least 37,5%

Weighted GSEA of EAT revealed 25 increased and 8 decreased biological processes in patients with persistent versus without AF (FDR adjusted p-value < 0.1) (Fig. 3). The majority of the increased processes (16/24) was associated withimmune system clusters and involved the closely related response to bacterium and neutrophil degranulation. These processesshare core proteins that are abundantly secreted by neutrophils, such as MPO, azurophil-1 (AZU1: FC N/A, p = 0.003) and elastase (ELANE: FC 16.5, p = 0.01). Cell surface interactions at the vascular wall featured the core protein integrin beta 2 (ITGB2: FC N/A, p = 0.007), which is crucial for leukocyte adhesion to the vascular wall prior to extravasation. Additionally, the increased reactive oxygen species metabolic process and hydrogen peroxide metabolic process involved core proteins such as acyl-co-enzyme A oxidase 1, the first enzyme of the fatty acid beta-oxidation pathway and producer of hydrogen peroxide (ACOX1: FC N/A, p = 0.002), and MPO, which forms a powerful oxidant upon reacting with hydrogen peroxide. Processes related to muscle contraction and heart development were decreased in EAT from patients with persistent AF compared to those without AF.

### Localization of MPO in EAT of patients with persistent and future onset AF

**Fig. 4:**
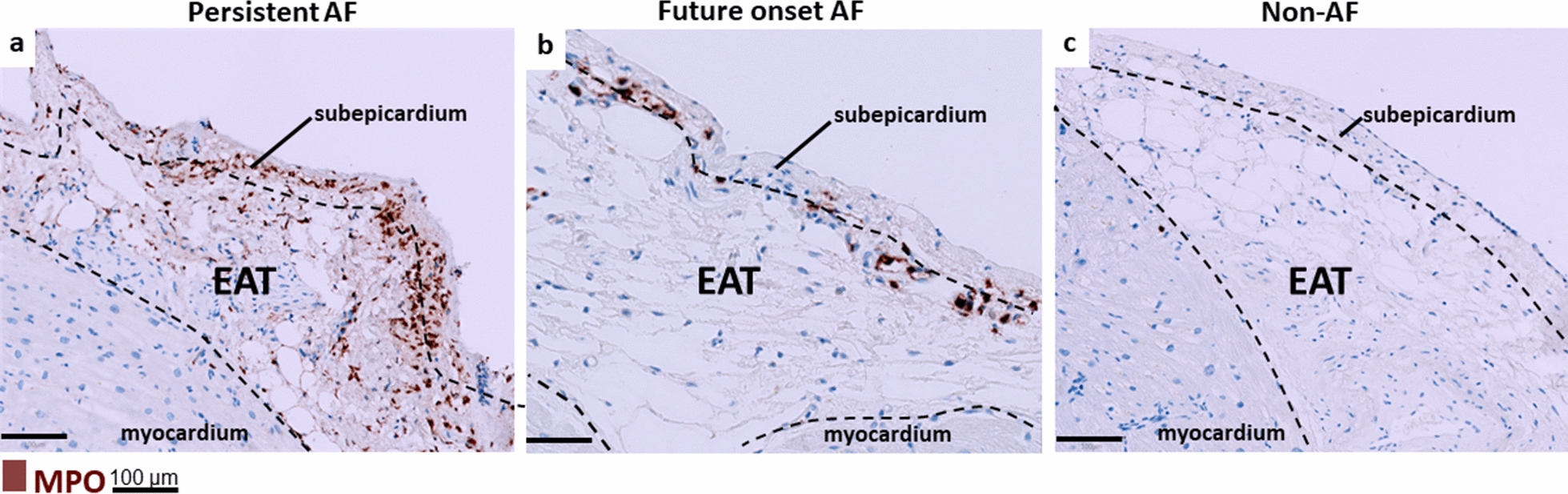
Sections of **a** persistent AF, **b** future onset AF, and **c** non-AF patients immunostained against MPO. Left: MPO positive cells show loss of well-defined cell structure, suggestive for neutrophil apoptosis and activation

**Fig. 5:**
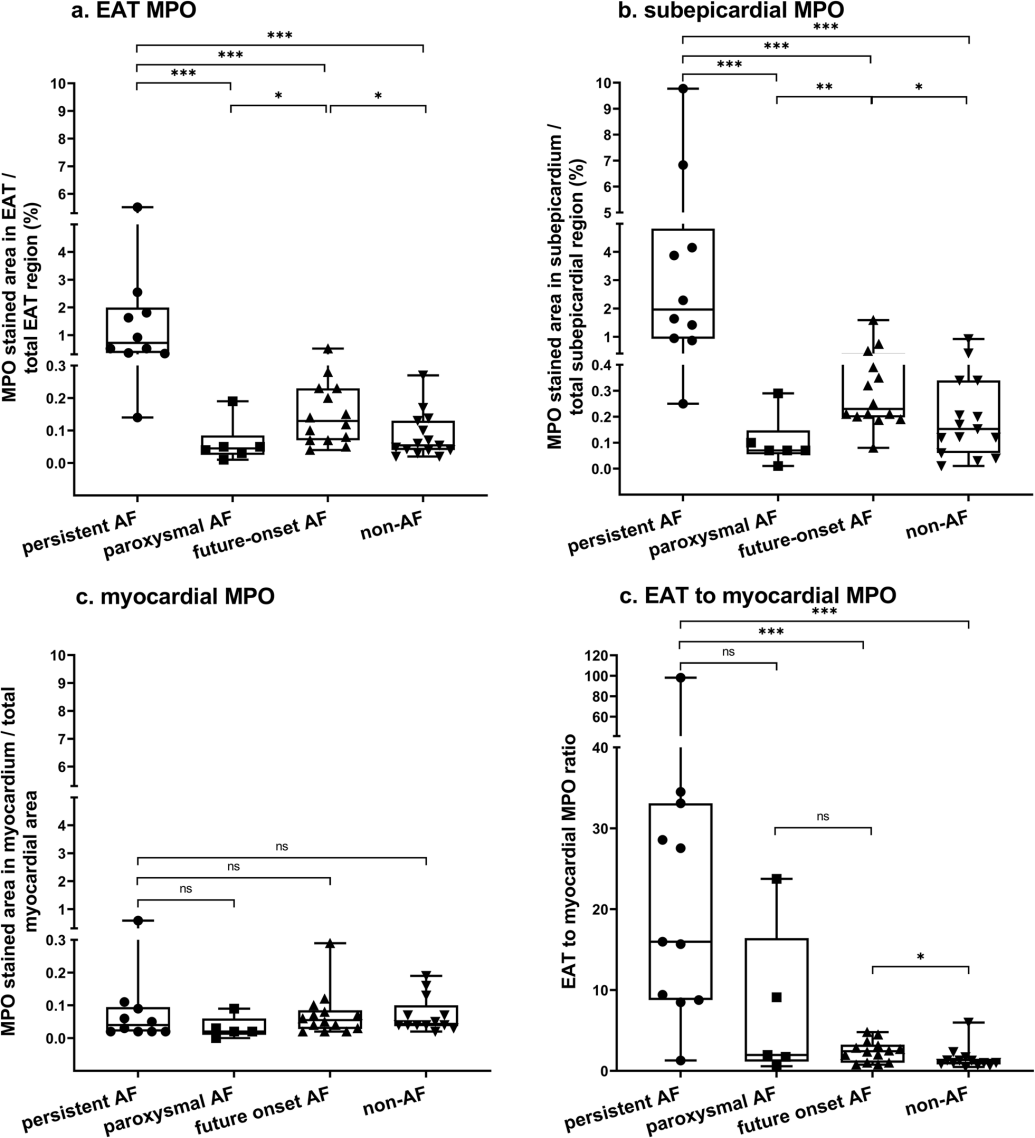
Percentages MPO of **a** the total epicardial adipose tissue (EAT), **b** the subepicardium specifically, **c** the myocardium. **d** shows the ratio MPO of EAT to myocardium. AF (persistent n = 10, paroxysmal n = 6), future onset AF (n = 15), and non-AF patients (n = 14) *p-value < 0.05, ** < 0.01, *** < 0.001; bars: median [IQR]

Total EAT and subepicardial MPO-stained areas were the largest in left atrial tissue in patients with persistent AF (FC 13.3, and FC 12.87, p < 0.0001) and were increased in patients with future onset AF (FC 2.4, p = 0.02, and FC 1.53, p = 0.03), compared to non-AF patients (Fig. 4, Fig. 5a, b). The EAT MPO-stained area was similar between paroxysmal and non-AF patients. TheMPO-stained area in the myocardium was similar across the groups (p = 0.26), and relatively smaller than that of the EAT (Fig. 5c, d). Interobserver variability (r) of MPO quantifications was 0.90, 0.89, and 0.95, with p ≤ 0.001 for total EAT, subepicardium, and myocardium, respectively.

MPO aggregations co-localized with fibrofatty strands that penetrated the myocardium in 70% ofpatients with persistent AF, in 33% with paroxysmal AF, in 27% with future onset AF, and in 7% of the non-AF patients (p = 0.01) (Additional file 1: Fig. S5). Generally, cells that positively stained for MPO were polymorphonuclear and negative for macrophage markers CD14, CD68 and CD163, suggesting that neutrophils, rather than macrophages, are the source of the identified MPO (Additional file 1: Fig. S6a,b). Furthermore, MPO was present inside and outside the vessels, indicating that at least a part of the MPO infiltrated the EAT (Additional file 1: Fig. S6a).

### Increased neutrophil extracellular traps in EAT from patients with persistent

#### AF

**Fig. 6:**
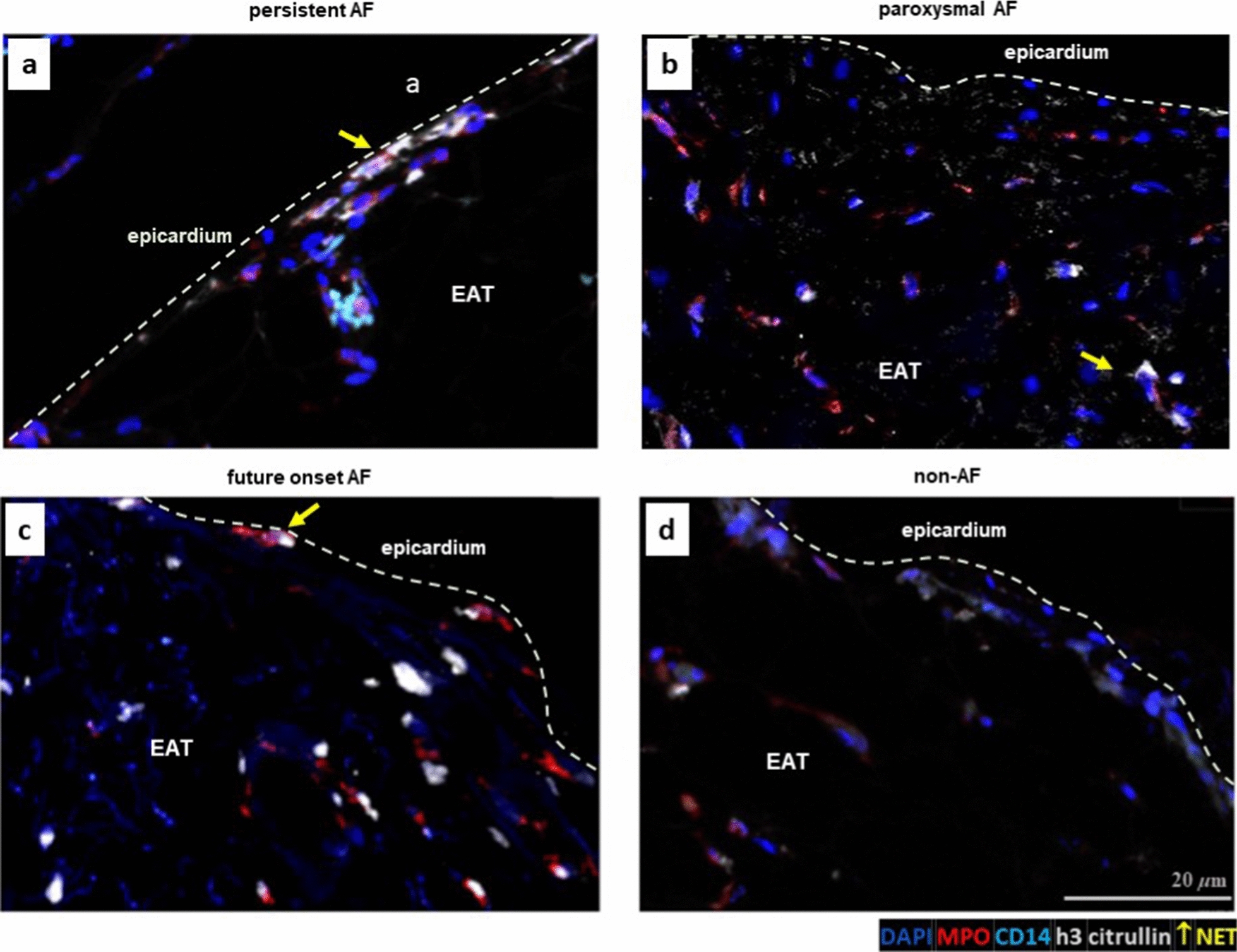
Neutrophil extracellular traps (NETs) in EAT from persistent AF **a**, paroxysmal AF **b**, future onset AF **c**, and non-AF **d** patients. NET producing neutrophils are those with web-like h3cit + structures that co-localize with MPO. CD31 + staining not shown

**Fig. 7:**
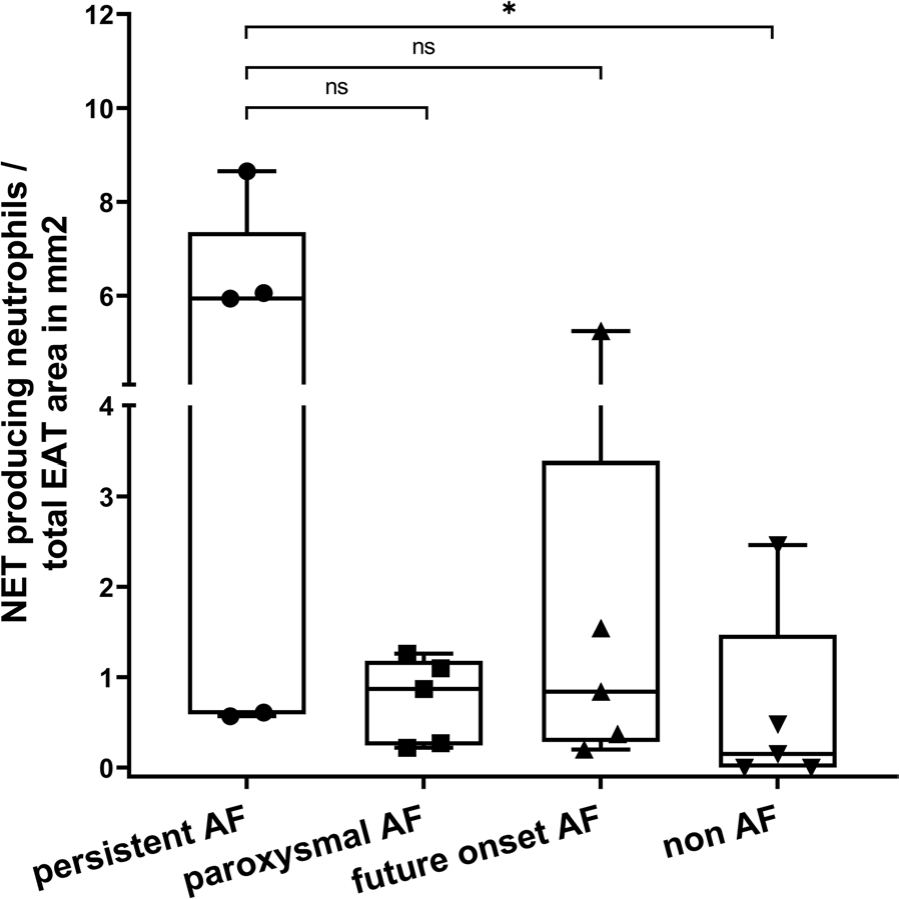
Bars represent neutrophil extracellular traps (NETs) in the EAT area of persistent, paroxysmal, future onset, and non AF patients (n = 5/group). NET counts were normalized to the total EAT area. Only extravascular NETs were counted. *p < 0.05; bars, median [IQR]

The number of extravascular neutrophil extracellular traps (NETs) was 39.3 times higher in the EAT from persistent compared to that of non-AF patients () but similar between paroxysmal AF and non-AF patients (FC5.8, p = 0.29) (Figs. 6 and 7). The number of NETs did not significantly differ between future onset and non-AF patients (FC 5.6, p = 0.21). Within the myocardium, in contrast to the EAT, only 2 NETs and 5 structures suspected as NETs could be identified across all patients.

## Discussion

This is the first explorative proteomics study on both the atrial EAT secretome and EAT from relatively healthy patients with AF and from more diseased patients without AF. We demonstrated that proteins abundant in neutrophil granules, such as MPO as well as proteins involved in coagulation were increased in the EAT secretome from patients with AF compared withpatients without AF who were older and had more cardiovascular risk factors. Supporting this, GSEA of the EAT proteome revealed increased neutrophil degranulation and oxidative stress in AF. Accordingly, MPO and neutrophil extracellular traps were upregulated in EAT from patients with persistent AF and appeared to aggregate into fibrofatty infiltrates within the atrial myocardium, structures associated with structural remodeling. Furthermore, among the patients without AF at baseline, EAT MPO was already increased in patients who would develop AF versus those who remained free of AF, while there were no differences in cardiovascular risk factors between these two groups. Altogether, these findings underscore an important association between EAT neutrophil activity and the fibrotic substrate of AF, irrespective of baseline differences between patient groups.

### The EAT secretome induces ECM gene expression in AF

ECM gene expression in atrial fibroblasts was more induced after exposure of the EAT secretome of patients with AF, compared to that of patients without AF (Fig. 1). This aligns with a previous study that reported increased stromal mesenchymal cell migration and fibrosis formation in rat ventricular tissue upon exposure to EAT vesicles from AF patients [36]. Here we utilized human atrial fibroblasts, which may be more clinically representative of atrial remodeling primarily driven by excessive extracellular matrix (ECM) deposition by fibroblasts. Patients with paroxysmal AF were younger than those with persistent AF. The EAT appears less profibrotic in paroxysmal AF patients (Additional file 1: Table S2), which could partially be explained by age as adiponectin and interleukin-6 gene expression were downregulated in EAT of old rats^27^. Conversely, the non-AF patients in this study were older but their secretome was less profibrotic than that of AF patients. Thus, it is presumable that age is not driving the differences in profibrotic effect of EAT. We conducted further experiments to examine the profibrotic activity of AF EAT secretome in cultures of neonatal rat ventricular myocytes (NRVM) mixed with fibroblasts (60 and 40%, respectively, based on flow cytometry). In these experiments, we observed that a 3-day incubation with human AF EAT secretome, compared to control medium (CCM), resulted in increased gene expression of the fibroblast enhanced genes: BGN, COL3A1, and MMP2 (Ernault et. al, data not shown).

### Profibrotic proteins secreted by EAT

MPO is a peroxidase enzyme primarily secreted by neutrophils. In the extracellular space, MPO induces fibroblast proliferation, migration, and ECM expression [10]. Furthermore, MPO activates matrix metalloproteinases (MMPs) and recruits neutrophils, which themselves are potent generators of MMPs and cytokines. Accordingly, MMP9 was highly increased in EAT of AF patients (Additional file 1: Table S3). MPO has been associated to poor outcomes in AF patients [2, 33]. Furthermore, MPO-deficient mice were protected from developing AF upon rapid pacing, which reversed after adding MPO [33]. Thus, MPO appears to play a role in the substrate of AF.

Other proteins increased in AF include those involved in coagulation. Coagulation factors, such as thrombin, have profibrotic and inflammatory effects within atrial tissue by increasing the expression of transforming growth factor beta 1 and monocyte chemoattractant protein-1, as demonstrated in isolated rat atrial fibroblasts [38]. Furthermore, thrombin inhibition in a goat model of AF led to reduced atrial activation time and increased endomysial fibrosis compared with untreated goats [24, 38]. Fibrinogen, which was increased in AF in this study, stimulates the proliferation of human fibroblasts [16]. These results suggests that EAT mayattract and serve as a reservoir for profibrotic proteins.

### Signs of increased neutrophil activity in EAT from patients with AF

The weighted GSEA revealed increased *neutrophil degranulation* and *cell surface interaction at the vascular wall,* suggesting increased neutrophil activity and immune cell extravasation in EAT from patients with compared to those without AF (Fig. 3) [6]. Also, *the hydrogen peroxidase metabolic process* was increased in AF. Oxidative stress induces tissue necrosis, which subsequently attracts (more) neutrophils [1]. Unweighted GSEA revealed that the *glycerolipid metabolism process* was increased. Immune cells, including neutrophils and adipocytes, secrete chemokines upon activation of the NF-kB pathway by hypoxia and free fatty acids (FFA), which in turn attract more neutrophils to the EAT [31, 44]. Unweighted GSEA further revealed *leukocyte migration*, *extracellular matrix and structure reorganization*, *coagulation*, and *regulation of epithelial cell migration* (data not shown). Increased *neutrophil degranulation* was supported by increased expression of most of these proteins in the secretome of persistent versus non-AF patients. However, the relative increase in proteins other than MPO was less pronounced in the EAT itself (Additional file 1: Tables S3, S4). This discrepancy may be explained by the fact that only 36% or fewer of human proteins are predicted to be secreted [40]. Nevertheless, the considerable increase in MPO in the AF EAT secretome emphasizes neutrophil secretion. Furthermore, neutrophils contribute to a pro-inflammatory environment via NETosis. Extracellular DNA material and co-aggregating proteins such as MPO attract leukocytes, maintaining and promoting the inflammatory state. Indeed, we found more EAT NETs in persistent than in non-AF patients (Figs. 6 and 7). Interestingly, coagulation factors, chemotactic for neutrophils that can promote thrombus formation by releasing NETs, were increased in both AF EAT secretome and EAT [15, 22].

Our findings are consistent with our previously reported increase of MPO and NETs in whole LAAs from persistent AF compared to non-AF patients. MPO levels were also shown to be increased in right atrial appendages (RAAs) from patients with compared to without AF.[20, 33]. However, at least in our study, fragments of EAT and fibrofatty infiltrates were still attached to the myocardium. It is plausible that even minimal contamination of the myocardial biopsies by small fragments of EAT and MPO strands infiltrating the myocardium could have influenced the MPO expression of the myocardium, given the significant overexpression of MPO in EAT from AF patients. To address this concern, we conducted an immunohistological analysis to investigate the distribution of MPO in the tissue. Here we found that the MPO aggregates primarily in the EAT but not in the myocardium. In contrast to EAT MPO, the relatively low presence of intramyocardial MPO did not differ between patients with and without AF. Therefore, the increase of MPO levels in patients with compared to those without AF is explained by MPO aggregation in the EAT rather than the myocardium. Interestingly, EAT MPO was already increased in patients who would develop AF during follow-up, compared to patients who never developed AF (matched for BMI and CHA_2_DS_2_VASc score; Fig. 4 and 5a), indicating that MPO may be implicated in AF development. Paatients with paroxysmal AF have less abundant MPO compared to those with persistent and future onset AF. This could be explained at least in part by (numerically) fewer comorbidities and lower age in paroxysmal AF patients or by development of persistent instead of paroxysmal AF in several future onset AF patients.

### MPO aggregates in the subepicardium and co-localizes with fibrofatty infiltrates

MPO was abundant in the subepicardial area of the EAT (Figs. 4 and 5b). Subepicardial MPO localizes in the area where epithelial-to-mesenchymal transition (EMT) occurs. In patients with atrial cardiomyopathy, EMT of epicardial cells induces fibroblast and adipocyte proliferation, contributing to fibrofatty infiltration of the subepicardium [39]. Furthermore, we recently reported EMT as an important mechanism underlying the formation of the fibrotic substrate in AF [41]. Interestingly, MPO-containing NETs were recently found to induce EMT in lung epithelial cells [28]. Accordingly, we found more NETs and increased *epithelial cell migration* in the EAT of patients with compared to those without AF. Whether MPO and NETs are involved in EMT in the setting of AF requires future research.

EAT MPO co-localized with fibrofatty strands infiltrating the myocardium and was most abundant in patients with persistent AF (Additional file 1: Fig. S5). Fibrofatty infiltrates are associated with AF, potentially through conduction slowing or heterogeneity [12, 17]. Besides myocardial infiltration, which potentially forms structural conduction barriers, we recently demonstrated a direct effect on conduction slowing and heterogeneity in cardiomyocyte cultures exposed to EAT secretome of patients with AF [13]. No conduction slowing was observed when cell cultures were exposed to subcutaneous AT secretome. It is plausible that fibrofatty MPO infiltrates are involved in structural remodeling by promoting ECM production from surrounding fibroblasts. Alternatively, such infiltrates might induce electrical remodeling through electrophysiological changes within or between myocytes.

### Clinical implications

Our novel finding is that EAT in patients with AF, compared to that of patients without AF, is a neutrophil-rich tissue that secretes numerous profibrotic molecules, including MPO, which is already present before the onset of AF. MPO primarily localizes around fibrofatty infiltrates, suggesting MPO may serve as a marker of structural remodeling in AF. A causal role of MPO in fibrosis formation in AF remains to be established. The activity of neutrophils, the primary source of MPO, can be inhibited by the anti-inflammatory drug colchicine, which has been shown to reduce AF recurrence after PVI [21, 43]. Additionally, colchicine showed a strong trend to decreased AF recurrences in patients with large LA EAT volumes undergoing AF ablation, while it did not affect the recurrence rate in patients with small EAT volumes [46]. This trend may be mediated by inhibiting EAT neutrophil activity. Furthermore, a specific MPO blocker (AZD4831) with anti-inflammatory and -fibrotic properties has been clinically applied in patients with heart failure with preserved ejection fraction. However, no data is available on the effect of MPO blockers in patients with AF [26]. Our findings suggest that EAT may play a role in the formation of the arrhythmogenic substrate in AF. Therefore, both neutrophils and MPO are promising targets for future research into prevention or therapy.

### Limitations

Most patients without AF had coronary artery disease (CAD), which is associated with locally increased neutrophil accumulation. However, EAT MPO was lower in patients without compared to those with AF. Patients without AF used statins, metformin, and antiplatelet therapy, all potential inhibitors of neutrophil activity, more often than AF patients, consistent with their clinical profile [8, 25]. Nevertheless, the blood leukocyte count was higher and the thrombocyte count was similar in patients without AF versus those with AF. In the proteomics cohort, MPO was increased in AF while metformin and statin use did not differ between patients with and without AF [20], and in the immunohistochemistry cohort, MPO was increased in future onset AF, while medication use, age, BMI, diabetes%, and LAVI did not differ between future onset and non-AF patients. Together, these data suggests that the increased MPO in the left atrial EAT of AF patients occurs independently from hematological effects, medication and comorbidities.

Increased EAT volume has been associated with AF. We did not correct for the area of EAT in histology because LAA section size differed between patients. The EAT was thoroughly washed with PBS but not perfused. Extravascular MPO and NETs could be distinguished by co-staining for endothelial CD31 (Additional file 1: Fig. S6a). Extravascular NETs were increased in persistent AF, indicating that intravascular MPO did not significantly affect our findings. The small size of the proteomics and NETs cohorts could have resulted in false-positive results. However, we immunohistochemically validated MPO abundance in 45 LAA sections and performed blinded for patient group and outcome.

## Conclusion

For the first time, we demonstrate that in atrial epicardial adipose tissue, MPO and NETs emerge as potential contributors to fibrosis formation in AF. Our findings indicate that MPO starts to accumulate prior to the clinical onset of AF and that the profibrotic effect of the EAT secretome of AF patients intensifies with AF progression. Altogether, this study provides insight into how EAT can facilitate fibrosis and highlights the significance of neutrophil activity in the pathophysiology of AF.

## Supplementary Information

**Additional file 1: Figure S1.** Gel imageand slicing schemeof EAT secretome and EAT samples. **Figure S2.** MPO quantification in predefined epicardial layers, explanatory figure. **Figure S3.** a, b. Unsupervised clustering of EAT secretome and EAT proteomes. **Figure S4.** Similar differentially expressed proteins in EAT, EAT secretome and myocardium. **Figure S5.** MPO co-localizes with fibrofatty strands that penetrate the myocardium in persistent AF patients. **Figure S6.** a, b. Extravascularly localized EAT MPO and NETs. **Table S1**. Gene primer-sets. **Table S2.** Top 50 most differentially expressed proteins in AF EAT secretome. **Table S3.** Top 50 most differentially expressed proteins in AF EAT. **Table S4.** Patient characteristics: EAT secretome - fibroblast culture study

## Data Availability

The proteomes from the EAT and the EAT secretome were uploaded to the ProteomeXchange Consortium via the proteomics identification database PRIDE with accession number PXD013230.

## Abbreviations

AF: Atrial fibrillation
BH: Benjamini-Hoghberg
DAB: 3,3'-diaminobenzidine
EAT: Epicardial adipose tissue
ECM: Extracellular matrix
EMT: Epithelial-to-mesenchymal transition
FC: Fold change
FFA: Free fatty acids
GSEA: Gene set enrichment analysis
LAA: Left atrial appendage
MMP: Matrix metalloproteinase
MAPK: Mitogen-activated protein kinases
SR: Sinus rhythm
