# Supplementary material for: Atrial epicardial adipose tissue abundantly secretes myeloperoxidase and activates atrial fibroblasts in patients with atrial fibrillation
Source: J Transl Med. 2023 Jun 6;21:366. doi: 10.1186/s12967-023-04231-2 (PMC10245533; doi:10.1186/s12967-023-04231-2)
Supplement: Supplementary file 1 — Additional file 1: Figure S1. Gel imageand slicing schemeof EAT secretome and EAT samples. Figure S2. MPO quantification in predefined epicardial layers, explanatory figure. Figure S3. a, b. Unsupervised clustering of EAT secretome and EAT proteomes. Figure S4. Similar differentially expressed proteins in EAT, EAT secretome and myocardium. Figure S5. MPO co-localizes with fibrofatty strands that penetrate the myocardium in persistent AF patients. Figure S6. a, b. Extravascularly localized EAT MPO and NETs. Table S1. Gene primer-sets. Table S2. Top 50 most differentially expressed proteins in AF EAT secretome. Table S3. Top 50 most differentially expressed proteins in AF EAT. Table S4. Patient characteristics: EAT secretome - fibroblast culture study [file 12967_2023_4231_MOESM1_ESM.pdf]

Supplementary data: Epicardial adipose tissue abundantly secretes myeloperoxidase and activates atrial fibroblasts in patients with atrial fibrillation

Supplemental figure 1. Gel image(s) and slicing scheme(s) of EAT secretome and EAT samples

Supplemental figure 2. MPO quantification in predefined epicardial layers, explanatory figure

Supplemental figure 3a,b. Unsupervised clustering of EAT secretome and EAT proteomes

Supplemental figure 4. Similar differentially expressed proteins in EAT, EAT secretome and myocardium

Supplemental figure 5. MPO co-localizes with fibrofatty strands that penetrate the myocardium in persistent AF patients

Supplemental figure 6a,b. Extravascularly localized EAT MPO and NETs

Supplemental table 1. Gene primer-sets

Supplemental table 2. Top 50 most differentially expressed proteins in AF EAT secretome

Supplemental table 3. Top 50 most differentially expressed proteins in AF EAT

Supplemental table 4. Patient characteristics: EAT secretome - fibroblast culture study

Figure 1. Gel image(s) and slicing scheme(s) of EAT secretome and EAT samples

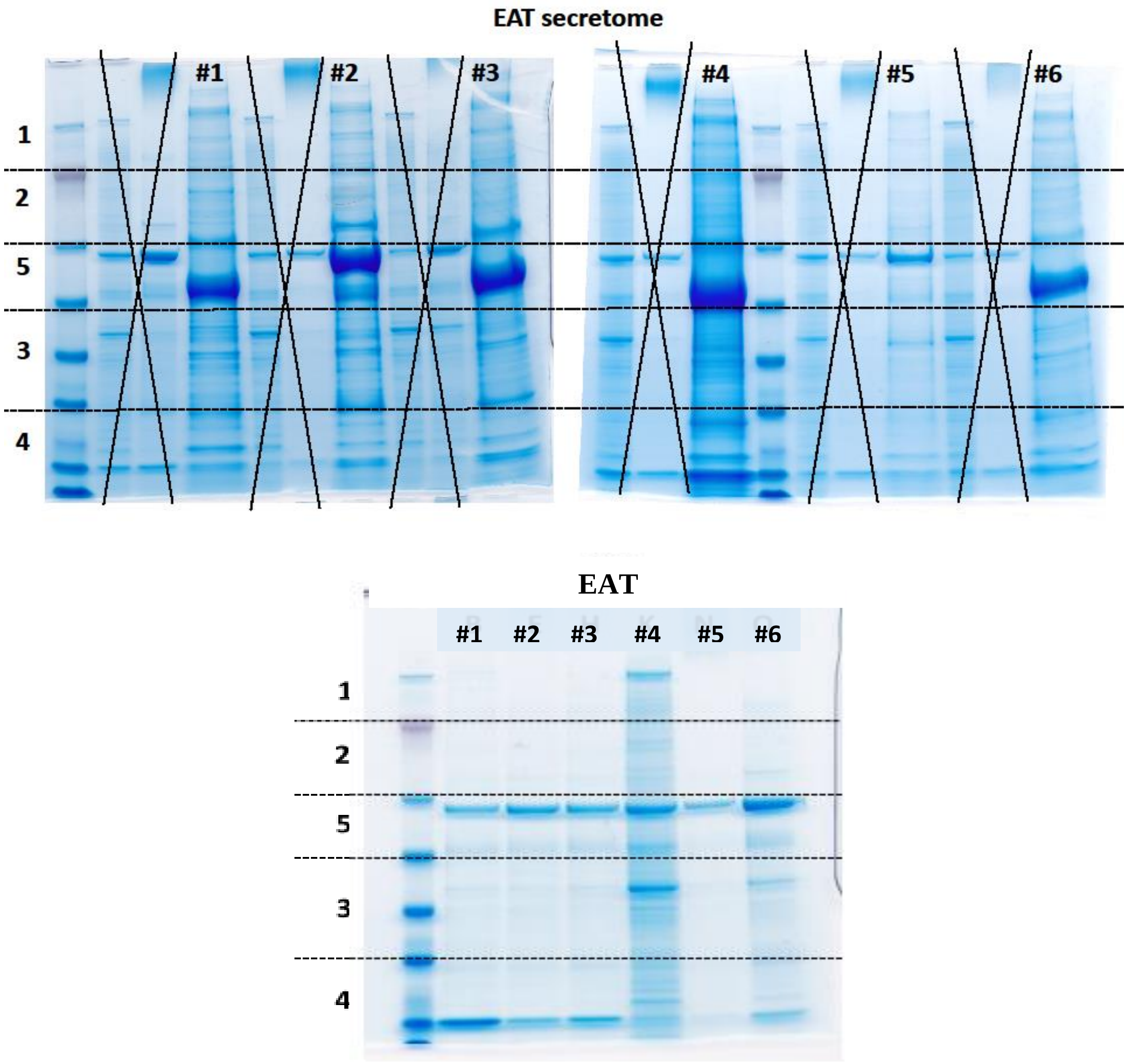

The marker was loaded in the first lane. In lane #1,2,3 samples from patients without, and in lane #4,5,6 samples from patients with persistent AF were loaded.

Figure 2: MPO quantification in predefined epicardial layers, explanatory figure

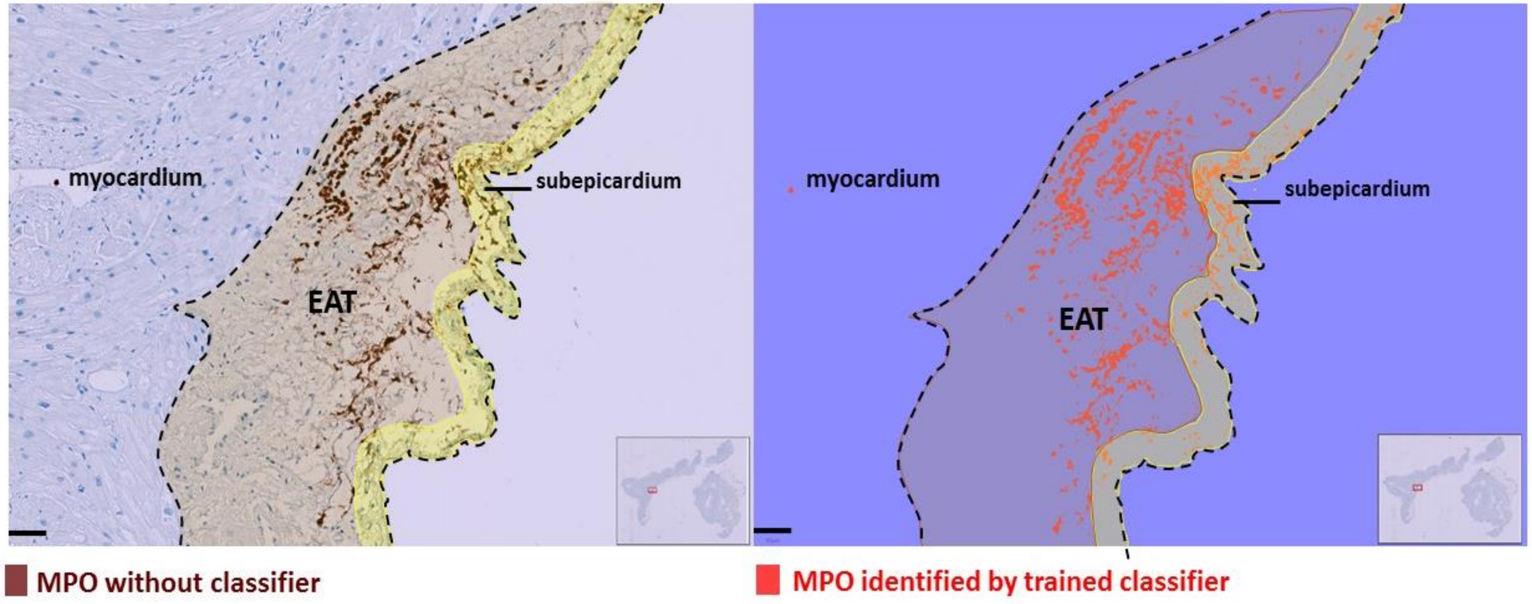

Immunohistochemical MPO staining in EAT from an AF patient. Left: MPO identification without trained classifier, right: identification with classifier. EAT, epicardial adipose tissue. Yellow: subepicardial region; orange: EAT

Figure 3a,b. Unsupervised clustering of EAT secretome and EAT proteomes

Figure 3a

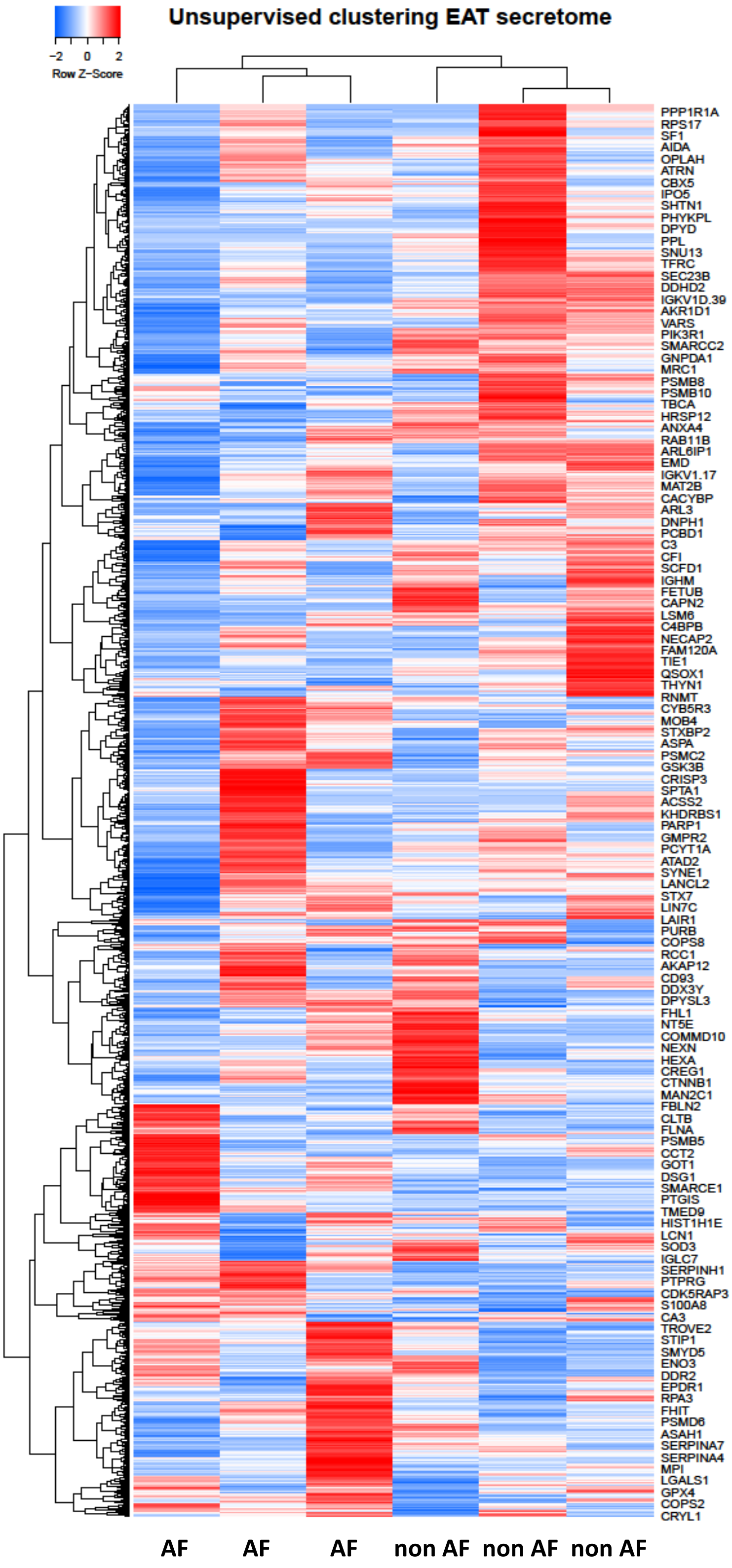

Figure 3b

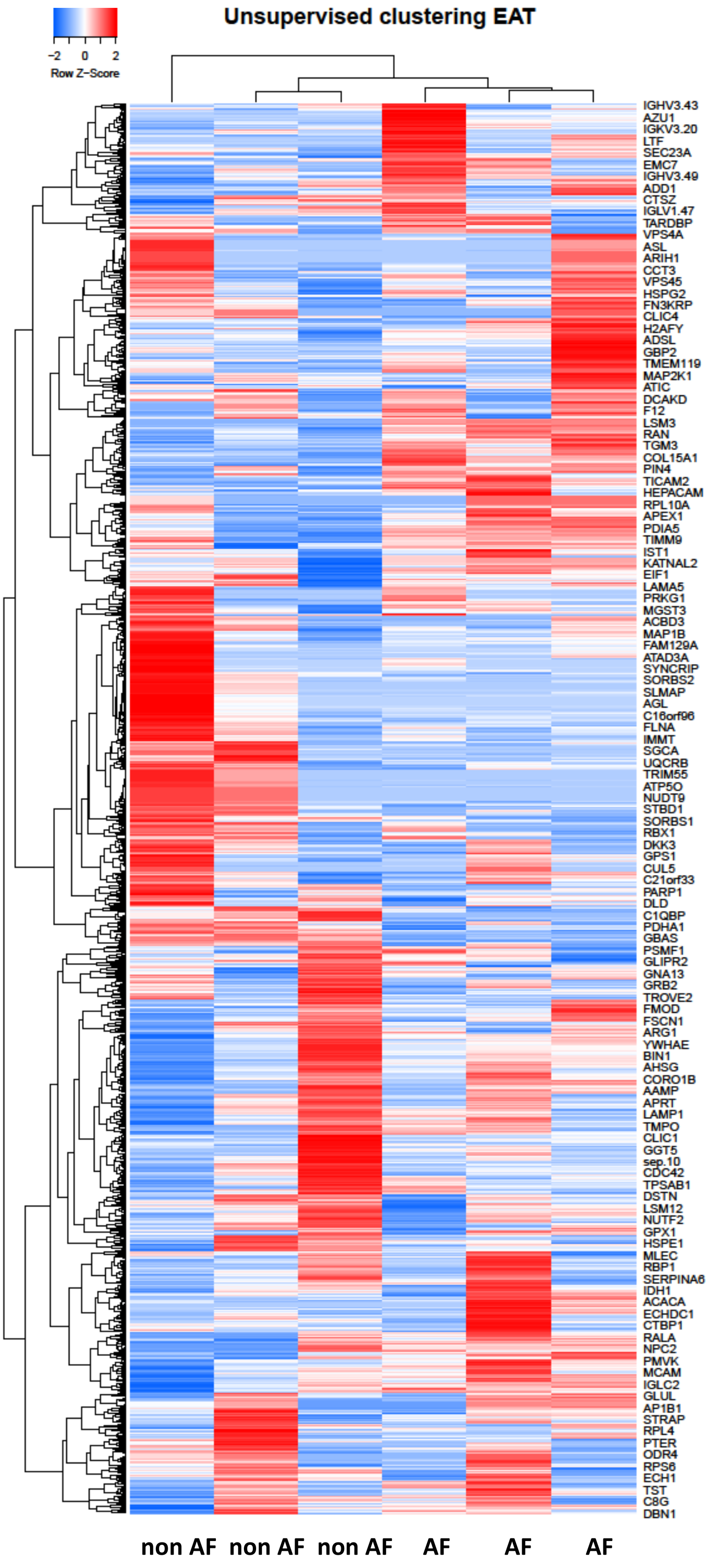

Unsupervised hierarchical clustering of all identified proteins (y-axis) and clustering of patient phenotype in EAT secretome (a) and EAT (b). Data is grouped by similar protein expression levels, calculated by average linkage with Pearson distance calculation. Heatmap colors represent relative increased or decreased protein expression, as indicated by the z score.

Figure 4. Similar differentially expressed proteins in EAT, EAT secretome and myocardium

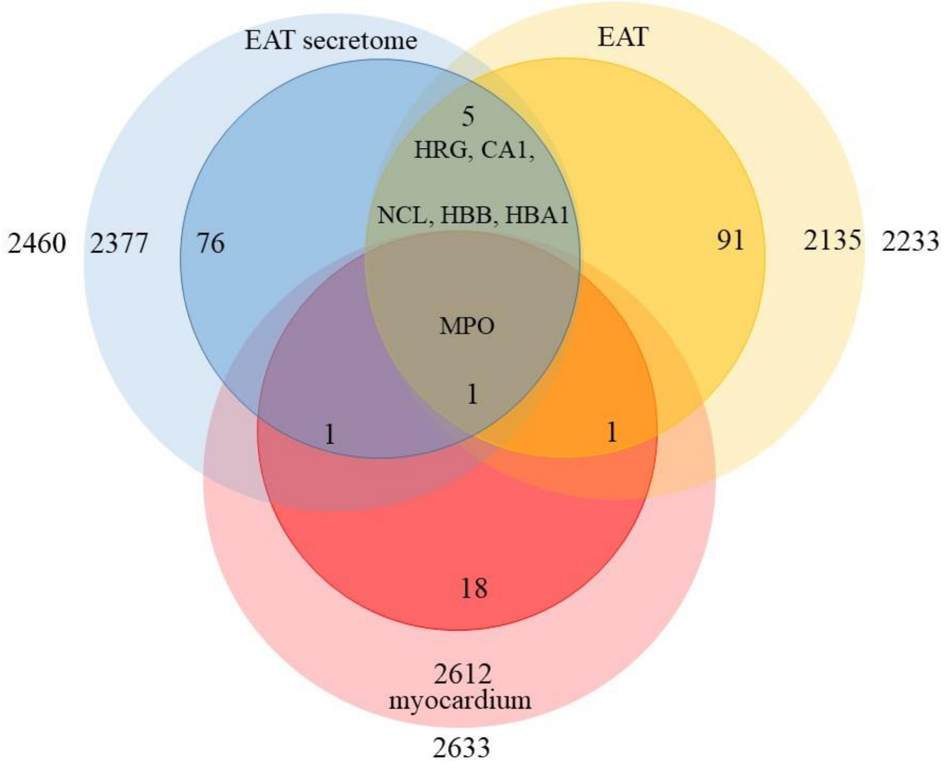

The smaller circles indicate differentially expressed proteins p<0.01 in EAT secretome (blue), EAT (yellow), and myocardium (red) of the same 6 patients, identified by LC-MS/MS. Six similar proteins were differentially expressed in both EAT secretome and EAT. All were increased in both EAT and in EAT secretome, except for NCL, nucleolin.
MPO was the only protein differentially expressed in AF versus non AF in all tissue types.
HRG, Histidine-rich glycoprotein; MPO, myeloperoxidase; CA1, carbonic anhydrase 1; HBB, hemoglobin subunit beta; HBA1, haemoglobin A1

Figure 5. MPO co-localization with fibrofatty strands that penetrate the myocardium

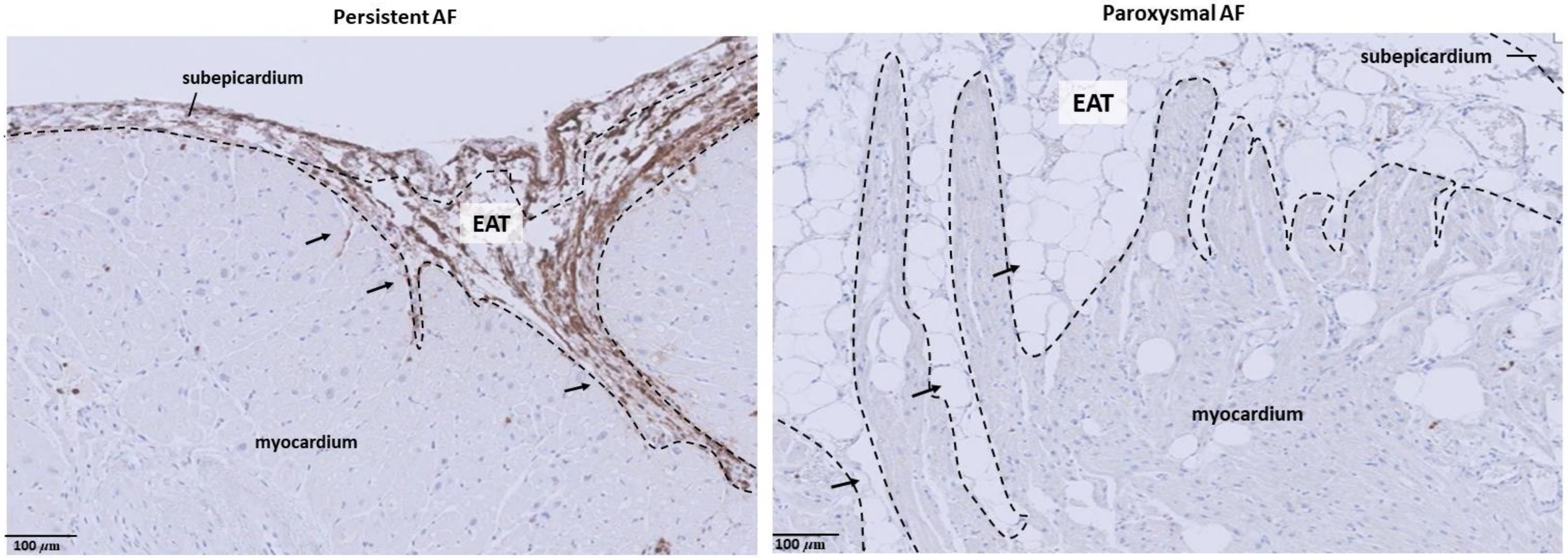

MPO co-localizes with fibrofatty strands that infiltrate into the myocardium in persistent AF (left) but not in paroxysmal AF (right). MPO-fibrofatty strands were present in 8/9 patients with persistent AF, in none with paroxysmal AF, in 5/15 with future onset AF and in 3/14 without AF. Arrows indicate fibrofatty infiltrates.

Figure 6a,b. EAT MPO source and NETs localization

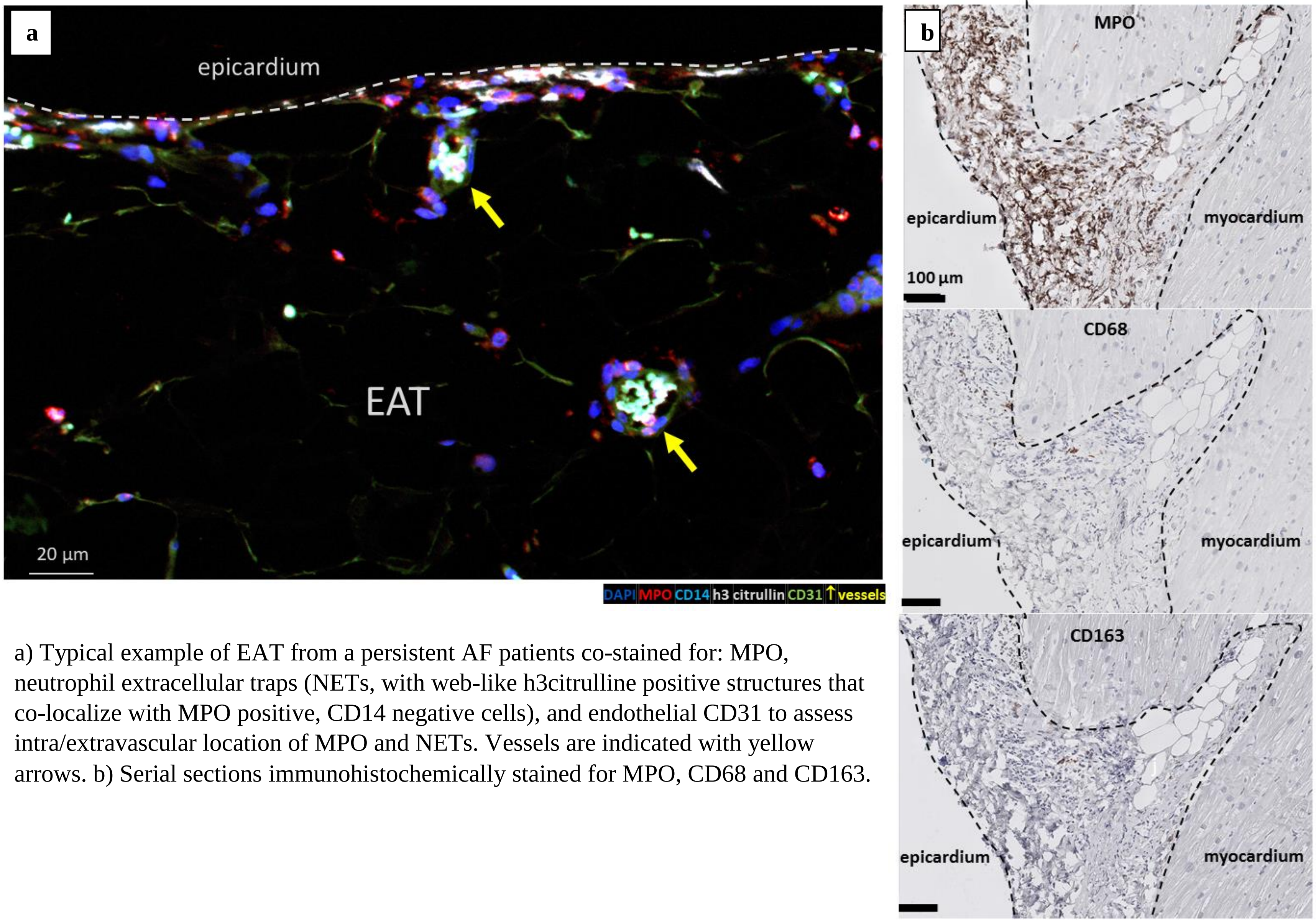

a) Typical example of EAT from a persistent AF patients co-stained for: MPO, neutrophil extracellular traps (NETs, with web-like h3citrulline positive structures that co-localize with MPO positive, CD14 negative cells), and endothelial CD31 to assess intra/extravascular location of MPO and NETs. Vessels are indicated with yellow arrows. b) Serial sections immunohistochemically stained for MPO, CD68 and CD163.

Table 1. Gene primer-sets

Gene	Forward	Reverse
hCOL1A1	5"-CTGAGCCAGCAGATCGAGAA-3",	3"-ATCCAGTASTCTCCACTCTTCCA-5"
hFN1	5"-CTGCAAGCCCATAGCTGAGA-3"	3"-GAAGTGCAAGTGATGCGTCC-5"
hHRPT	5-"CCCTGGCGTCGTGATTAGTGA-3"	3"-"AGAGGGCTASAATGTGATGGC-5"

Table 2. Patient characteristics: EAT secretome - fibroblast culture study

Variable	Persistent AF (n=11)	Paroxysmal AF (n=3)	Without AF (n=6)	p-value
**Surgery type**	Thoracoscopic AF ablation	Thoracoscopic AF ablation	CABG	N/A
**Male sex n (%)**	11 (100)	3 (100)	6 (100)	1.0
**Age [IQR]**	63.0 [11.0]	54.0 [N/A]	70.0 [7.5]	0.04
**BMI (±SD)**	28.8 (3.5)	28.7 (2.7)	27.75 (1.6)	0.77
**LAVI % [IQR]**	41.9 [22]	29.3 [NA]	33.0 [10]	<0.01*
**LVEF% [IQR]**	54.1 [6]	NA	55.7 [24]	0.63*
**CHA_2DS_2-VASc [IQR]**	1 [2]	0 [2]	3 [2]	0.02
**Hypertension n (%)**	3 (27)	1 (33)	3 (50)	0.83
**Diabetes n (%)**	0 (0)	1 (33)	4 (67)	<0.01
**Vascular disease n (%)**	0 (0)	0 (0)	6 (100)	<0.01
**Stroke n (%)**	2 (18)	0 (0)	1(17)	0.73
**Congestive heartfailure n (%)**	1 (9)	0 (0)	0 (0)	0.65
**CRP [IQR]**	1.7 (2.2)	0.8 [NA]	0.4 [15.6]	0.69
**Leukocytes 10–9/L (±SD)**	7.8 (0.20)	6.43 (1.62)	8.12 (1.86)	0.16
**Thrombocytes 10–9/L (±SD)**	260.18 (42.44)	219.33 (16.62)	262.6 (43.63)	0.28
**OAC n (%)**	11 (100)	3 (100)	0 (0)	<0.01
**Antiplatelet n (%)**	0 (0)	0 (0)	6 (100)	<0.01
**Statins n (%)**	1 (9)	1 (33)	6 (100)	<0.01

CABG, coronary artery bypass grafting; BMI, body mass index, LAVI, left atrial volume index; LVEF, left ventricular ejection fraction; NA, not available; N/A, not applicable; OAC, oral anticoagulant; PAF, paroxysmal AF; peAF, persistent AF; *t-test between peAF versus non-AF instead of three group ANOVA due to low number of PAF patients.

Table 3. Top 50 most differentially expressed proteins in AF EAT secretome

protein name	peAF	peAF	peAF	non AF	non AF	non AF	fold change	p-value	BH corrected p-value 0,25
ACADVL	2	6	1	0	0	0	N/A	0,0056	0,0049
MAOA	1	3	2	0	0	0	N/A	0,0068	0,0062
MPO	26	19	8	0	0	3	18,08	0,0026	**0,0028**
ADSSL1	4	7	3	2	0	0	8,25	0,0055	0,0048
EIF4G2	6	3	5	0	2	0	6,60	0,0075	0,0069
CKAP4	6	6	5	3	0	0	6,02	0,0068	0,0060
ALDH7A1	5	6	7	0	2	1	5,90	0,0029	**0,0030**
ECH1	7	10	3	0	2	2	5,10	0,0080	0,0072
GLUD1	11	19	8	4	2	3	3,99	0,0030	**0,0032**
AIFM1	10	7	5	2	1	3	3,82	0,0066	0,0058
CA2	23	14	17	5	5	6	3,33	0,0006	**0,0011**
CA1	67	40	57	18	13	20	3,18	0,0003	**0,0005**
SERPINH1	9	7	7	2	4	2	2,99	0,0068	0,0063
EPHX1	17	20	13	10	4	4	2,78	0,0043	0,0037
PSMD12	9	12	14	7	2	4	2,63	0,0087	0,0077
HBD	40	33	37	13	12	21	2,39	0,0006	**0,0012**
HBB	488	526	687	220	193	315	2,34	0,0007	**0,0013**
HBA1	440	368	432	135	161	247	2,29	0,0004	**0,0007**
HSPA1B	160	216	158	88	72	99	2,06	0,0005	**0,0009**
ILK	41	27	31	16	13	20	2,00	0,0029	**0,0029**
HRG	67	59	65	31	30	36	1,98	0,0001	**0,0004**
VTN	30	26	23	13	15	17	1,73	0,0054	0,0045
AHCY	26	32	31	16	19	17	1,71	0,0049	0,0042
ANXA11	30	24	27	15	17	19	1,60	0,0091	0,0080
HSPA8	206	233	171	150	105	143	1,53	0,0064	0,0057
HSPG2	235	226	211	143	159	155	1,47	0,0001	**0,0003**
GSN	161	127	123	180	174	189	-1,32	0,0061	0,0054
VCP	135	147	133	182	189	177	-1,33	0,0006	**0,0010**
NCL	57	75	49	88	100	80	-1,48	0,0052	0,0044
C3	707	367	576	865	944	900	-1,64	0,0063	0,0055
GDI2	35	39	51	69	76	65	-1,67	0,0011	**0,0017**
CFB	105	62	71	144	136	146	-1,79	0,0014	**0,0019**
MAP4	45	22	41	74	52	69	-1,81	0,0099	0,0083
SERPINF1	19	17	14	40	22	31	-1,88	0,0095	0,0082
KLKB1	22	12	13	28	31	27	-1,88	0,0059	0,0052
TAGLN2	19	22	19	35	46	33	-1,91	0,0014	**0,0020**
CAPN2	13	9	9	27	16	19	-2,03	0,0092	0,0081
RRBP1	70	40	51	135	107	101	-2,12	0,0011	**0,0016**
COPB2	13	4	15	18	24	25	-2,12	0,0082	0,0074
ITIH4	67	24	40	72	108	100	-2,13	0,0100	0,0085
ENO1	83	104	86	191	225	185	-2,21	0,0000	**0,0002**
C2	21	7	10	31	28	29	-2,31	0,0045	0,0038
CD14	7	4	7	13	15	14	-2,31	0,0051	0,0043
SMARCC2	8	4	3	16	12	13	-2,53	0,0056	0,0050
LRG1	5	12	9	20	21	24	-2,54	0,0011	**0,0018**
AEBP1	9	4	3	17	16	11	-2,58	0,0078	0,0071
SERPINB9	7	3	5	9	16	12	-2,60	0,0088	0,0078
DBN1	12	3	7	24	15	18	-2,64	0,0077	0,0070
SEC23A	13	6	5	22	24	17	-2,72	0,0038	0,0036
AP1B1	5	1	7	15	13	10	-2,86	0,0046	0,0040

Proteins were first hierarchically listed based on p-value, thereafter according to fold change. N/A, not applicable: indicates only expressed in either AF or non-AF phenotype. Significant Benjamini-Hoghberg corrected p-values (FDR0,25) are presented in **bold**.

Table 4. Top 50 most differentially expressed proteins in AF EAT

protein name	peAF	peAF	peAF	non AF	non AF	non AF	fold change	p-value	BH corrected p-value 0,25
SLC27A6	0	0	0	3	4	7	-N/A	0,0013	**0,0024**
HSD17B8	0	0	0	4	2	7	-N/A	0,0015	**0,0026**
GORASP2	0	0	0	1	4	5	-N/A	0,0034	**0,0054**
SGCA	0	0	0	7	0	6	-N/A	0,0086	**0,0096**
SHTN1	0	0	0	4	0	3	-N/A	0,0094	**0,0104**
CD5L	7	3	4	0	0	0	N/A	0,0009	**0,0015**
IGKV1-6	5	5	3	0	0	0	N/A	0,0009	**0,0016**
MELTF	3	5	4	0	0	0	N/A	0,0013	**0,0022**
TMED4	4	3	3	0	0	0	N/A	0,0018	**0,0030**
ACOX1	5	2	4	0	0	0	N/A	0,0024	**0,0039**
HBQ1	4	2	6	0	0	0	N/A	0,0026	**0,0041**
TWF1	3	3	3	0	0	0	N/A	0,0030	**0,0044**
AZU1	21	3	6	0	0	0	N/A	0,0032	**0,0049**
MMP9	51	3	10	0	0	0	N/A	0,0045	**0,0060**
DMTN	1	3	4	0	0	0	N/A	0,0045	**0,0062**
MPP1	3	2	3	0	0	0	N/A	0,0064	**0,0076**
AKT2	3	3	1	0	0	0	N/A	0,0064	**0,0077**
PCK1	1	3	3	0	0	0	N/A	0,0065	**0,0078**
ITGB2	18	2	4	0	0	0	N/A	0,0069	**0,0081**
IGLV2-14	4	2	1	0	0	0	N/A	0,0082	**0,0093**
OLFM4	17	2	3	0	0	0	N/A	0,0089	**0,0100**
RAB27A	7	2	1	0	0	0	N/A	0,0091	**0,0103**
SPTA1	56	14	86	4	0	0	38,98051894	0,0051	**0,0071**
MPO	148	42	52	3	4	4	21,36705511	0,0047	**0,0064**
LCN2	25	5	16	1	0	1	21,08580948	0,0081	**0,0092**
RDH5	4	8	9	1	0	0	15,30661973	0,0021	**0,0034**
NQO2	9	15	13	3	0	1	10,75696085	0,0005	**0,0009**
ANPEP	4	5	3	1	0	0	8,589389036	0,0099	**0,0110**
SLC2A1	5	3	7	1	0	1	7,226525319	0,0080	**0,0091**
PTGES3	5	6	4	3	0	0	5,911224944	0,0099	**0,0109**
KPRP	5	3	4	0	2	0	5,653900541	0,0086	**0,0095**
HRG	31	20	15	5	4	3	5,34856048	0,0012	**0,0020**
PML	10	12	9	3	0	3	5,240963876	0,0020	**0,0032**
TGM3	7	3	9	1	2	0	5,155304095	0,0096	**0,0107**
RETSAT	18	17	19	5	4	3	4,119833808	0,0004	**0,0006**
IGHM	93	67	72	51	4	6	3,809757947	0,0060	**0,0072**
PYGL	21	21	24	7	4	7	3,690552398	0,0003	**0,0004**
POR	17	12	13	5	2	5	3,37324035	0,0023	**0,0038**
SH3GLB1	7	6	9	1	4	1	3,224566927	0,0095	**0,0105**
CA1	94	86	143	44	45	13	3,195566218	0,0038	**0,0056**
F13A1	41	30	61	20	9	14	3,046686561	0,0032	**0,0050**
ACSL1	147	259	179	96	54	62	2,763229013	0,0021	**0,0035**
HBB	1033	858	1049	498	463	122	2,714733367	0,0039	**0,0057**
IQGAP1	34	41	55	11	16	23	2,604611704	0,0031	**0,0046**
HSPA12A	26	24	31	13	9	10	2,520469844	0,0010	**0,0018**
HBA1	781	726	941	495	360	135	2,472575705	0,0048	**0,0067**
A2M	240	196	290	133	110	51	2,463911023	0,0033	**0,0052**
ITIH1	37	32	35	15	4	23	2,444318184	0,0068	**0,0079**
SERPING1	38	32	31	15	11	18	2,322310824	0,0010	**0,0017**
ACTR3	17	17	24	9	9	8	2,220283362	0,0051	**0,0069**

Proteins were first hierarchically listed based on p-value, thereafter according to fold change. N/A, not applicable: indicates only expressed in either AF or non-AF phenotype. Significant Benjamini-Hoghberg corrected p-values (FDR0,25) are presented in **bold.**
